# Assessing the Impact and Equity of an Integrated Rural Sanitation Approach: A Longitudinal Evaluation in 11 Sub-Saharan Africa and Asian Countries

**DOI:** 10.3390/ijerph17051808

**Published:** 2020-03-10

**Authors:** Paschal A. Apanga, Joshua V. Garn, Zoe Sakas, Matthew C. Freeman

**Affiliations:** 1 School of Community Health Sciences, University of Nevada, Reno, NV 89557, USA; papanga@unr.edu (P.A.A.); jgarn@unr.edu (J.V.G.); 2 Gangarosa Department of Environmental Health, Rollins School of Public Health, Emory University, Atlanta, GA 30322, USA; zoe.marie.sakas@emory.edu

**Keywords:** sanitation, coverage, equity, WASH, vulnerable

## Abstract

Few rural sanitation programs have documented large increases in sanitation coverage or have assessed if interventions equitably increase sanitation coverage for vulnerable groups. We characterize the impact of the Sustainable Sanitation and Hygiene for All (SSH4A) approach on key program WASH (water, sanitation, and hygiene) indicators, and also assess if these increases in WASH coverage are equitably reaching vulnerable groups. The SSH4A approach was administered in 12 program areas in 11 countries, including Bhutan, Ethiopia, Ghana, Indonesia, Kenya, Mozambique, Nepal, South Sudan, Tanzania, Uganda, and Zambia. Repeated cross-sectional household surveys were administered over four rounds at annual follow-up rounds from 2014 to 2018. Surveys were conducted in an average of 21,411 households at each round of data collection. Overall, sanitation coverage increased by 53 percentage points between baseline and the final round of data collection (95% CI: 52%, 54%). We estimate that 4.8 million people gained access to basic sanitation in these areas during the project period. Most countries also demonstrated movement up the sanitation ladder, in addition to increases in handwashing stations and safe disposal of child feces. When assessing equity—if sanitation coverage levels were similar comparing vulnerable and non-vulnerable groups—we observed that increases in coverage over time were generally comparable between vulnerable groups and non-vulnerable groups. However, the increase in sanitation coverage was slightly higher for higher wealth households compared to lower wealth households. Results from this study revealed a successful model of rural sanitation service delivery. However, further work should be done to explore the specific mechanisms that led to success of the intervention.

## 1. Introduction

Sustainable Development Goal (SDG) Target 6.2 aims to achieve access to adequate and equitable sanitation and hygiene for all and to end open defecation by 2030, yet 2.3 billion people still lack basic sanitation services and few countries are on track to achieve universal coverage [1,2,3]. Equity of sanitation access for vulnerable groups is a critical priority [1]. Achieving sanitation for all will require sanitation interventions that are able to reach marginalized or vulnerable groups. 

A recent systematic review found that previous sanitation interventions have only had modest impacts on increasing sanitation coverage and use [4]. Sub-optimal increases in coverage may limit the health benefits of sanitation programs [5,6,7,8]. While there is some consensus on the overall principles needed to achieve and sustain universal sanitation coverage [9], there is little rigorous evidence of successful programs at scale [10]. Context plays an important role in determining the implementation approach, so evidence of success and challenges of cross-country programs could support the rural sanitation and hygiene sector in achieving universal coverage. 

Inequity in sanitation provision remains a critical challenge, and there is little documentation of programs that successfully reach vulnerable populations. Studies have reported barriers with persons with disabilities and female-headed households in accessing water, sanitation, and hygiene (WASH) facilities [11,12], disparities in latrine coverage between elderly and younger persons [13], challenges for female headed households to obtain toilets both due to difficulty digging and toilet cost [12,14], and difficulties for poorer households to be able to afford the costs of building and maintaining a latrine [15]. The SSH4A approach targets universal access to sanitation with a focus on vulnerable, marginalized groups supported by gender and social inclusion strategies [16]. The approach also applies inclusive and pro-poor sanitation business models appropriate to the context of communities, and supports latrine builders and households on making informed choices on inclusive toilet designs [16]. 

SNV Netherlands Development Organization (SNV) and partners developed and implemented the Rural Sustainable Sanitation and Hygiene for All (SSH4A) approach, an integrated capacity building model with duty bearers (i.e., that national and local governments) focusing on: (1) demand creation, (2) sanitation supply chain and financing strengthening, (3) hygiene behavioral change communication, and (4) WASH governance. The multi-dimensionality of the SSH4A approach is meant to address barriers and more effectively scale sanitation interventions in a variety of contexts. This study evaluated the impact and equity of the SSH4A approach as it was implemented over a four-year period in 11 countries. Our primary aims were to characterize the program’s impact on increasing latrine coverage and to assess the equity in the levels of coverage for vulnerable or traditionally marginalized groups—such as persons with disabilities, elderly persons, households within the lowest wealth quintiles (i.e., lower socio-economic status) and female-headed households. Secondary aims included characterizing the program’s impact on moving households up the sanitation ladder, presence of a handwashing facilities at home, and safe disposal of child feces. Results from this study set a benchmark for improved coverage and equity of rural sanitation at scale and across country contexts and support adaptions to improve program performance. While this study focused on quantifying the impacts of the intervention on key WASH variables, this study did not explore the mechanisms for why and how the SSH4A approach might work. 

## 2. Materials and Methods

### 2.1. Study Context

Repeated cross-sectional household surveys were administered over four rounds at annual follow-up rounds from 2014 to 2018. The approach was administered in 12 program areas in 11 countries, including Bhutan, Ethiopia, Ghana, Indonesia, Kenya, Mozambique, Nepal, South Sudan, Tanzania, Uganda, and Zambia. The approach was administered in two program areas in Nepal (denoted as Nepal 1 and Nepal 2 in this paper), funded by different international donors. The SSH4A approach was implemented through the local government in all the countries involved. SSH4A is a capacity development approach that aims to strengthen key functions to enable sustainable sanitation. If there was already a sanitation focused initiative/program through the government, SNV would work with the government in the localities where SSH4A was rolled out and work within the government programming. If another non-governmental organization (NGO) was working in the same area where SNV wanted to work, reflection meetings were held to choose separate localities in which to work; other sites were always chosen if this issue arose. The surveys were administered by SNV in each program area, and the surveys consisted of common data collection methods that were overseen by central monitoring and evaluation specialists. The data were externally verified in nine of the eleven countries (because they were part of a results-based finance arrangement, required by the donor funding the project in those countries). 

### 2.2. SSH4A Approach

The SSH4A approach was developed by SNV and other partners starting in 2008, in partnership with government line agencies, and subsequently tested in five countries in Asia. Annual learning cycles and comparative studies among teams and partners have continued to iterate on the SSH4A approach. SSH4A has now been implemented across a total of 18 countries in Africa and Asia strengthening the implementation of government run programs. It focuses on capacity building primarily of government line agencies as duty bearers, including creating space for the development of local leadership, tailoring social mobilizing outreach mechanisms, and creating sustainable processes for demand creation and behavior change delivery steered by the local government workers. The SSH4A approach was integrated in local government planning and budgeting with the intention that activities would be sustained after SNV programs [16]. 

The approach aimed to increase area-wide rural sanitation coverage using an integrated model focusing on strengthening capacities around four components: (1) demand creation, (2) sanitation supply chain and financing strengthening, (3) hygiene behavioral change communication, and (4) WASH governance [16]. Adaptation of the SSH4A approach varies depending on local and national context within each country. Each program area experienced unique challenges and opportunities related to their service delivery. Extensive country-specific programmatic modifications and focus points are discussed in the appendix (Appendix A Text 1). The general description of the SSH4A components are reviewed below:

(1) *Demand creation* focuses on the capacity of local organizations to implement and steer sanitation demand creation processes at scale with quality in their area. That starts with the capacity of the local government to organize demand creation, ensuring harmonization, quality standards and sufficient attention to potentially vulnerable and/or culturally different groups. The component also includes strengthening of individual capacities of facilitators or health promotors to implement demand creation methodologies (often community-led total sanitation, or CLTS) respectfully, planned and in an inclusive way. It emphasizes timely post-triggering and follow-up, as well as support to informed technology choices by households.
(2) *Sanitation supply chain and financing strengthening* applies consumer studies, sanitation supply chain analysis, and business modelling to understand supply and demand of the sanitation market in each program area. Governments used this information to increase local capacity, improve financing mechanisms, support informed choice, and private sector to realize market-based solutions that meet changing consumer needs and preferences. Subsidies were not part of the SSH4A approach and emphasis was on the importance of local business development to ensure sustainability and community level support mechanisms. Some activities related to supply chain strengthening include hardware option analysis, development of informed choice materials for households (especially elderly and disabled), design of innovative latrine options, development of technology options handbooks, development of marketing materials, business development training for local businesses, masons communication training, review of affordability of latrines, and development of toilet upgrading strategies (e.g., adding a second pit or handwashing facility).
(3) *The hygiene behavioral change communication component* aimed at strengthening the capacity and implementation of evidence-based behavioral change communication for relevant agencies (partner agencies for this component in each country, depended on which agencies had the mandate for hygiene behaviour change activities within the country) at the sub-national level. The component targeted key hygiene behaviors emerging from the survey data such as cleanliness of toilets, using soap while hand washing, disposal of child feces. The behavior change methodology begins with stock taking of existing behavioral change activities and reflection on results so far, together with the responsible agency. Then it defines priority behaviors and audiences on which formative research is conducted. Application of findings from formative research studies, alongside participatory review of existing information, leads to the development of a locally-specific behavior change strategy (typically district level BCC strategies linked to the district sanitation strategy) and buy-in. Other activities include design of behavioral change communication (BCC) campaigns and other materials (e.g., posters, videos, radio messages, personal communication, activities, theater), training of field staff specific to BCC work (once individuals or local unit is assigned as responsible for BCC activities), and regular review and updating of the strategy, messages, and materials as needed.
(4) *WASH Governance* works to strengthen local authorities and support them to promote and achieve district-wide coverage. SNV engaged both locally (i.e., strengthening capacity for sustainable service delivery in local government, the private sector, and civil society) and at the sub-national level (i.e., working with the government, rights group holders and development partners to support sector reform). The WASH governance approach was based on the belief that national and local governments are the duty bearers for a progressive realization of sanitation as a human right in their countries and districts. Building capacity and leadership from the beginning with prioritized high-level governance was important to support the sustainability and scalability of WASH interventions. Some specific activities related to governance include regional workshops for decision makers and stakeholders, development of Open Defecation Free status (ODF) and post-ODF strategies and certification standards that all stakeholders agree with, dialogue with rights holder groups, development of pro-poor policies and mechanisms to support those in the lowest wealth quintile, targeted support for female-headed households, persons with disabilities, and the elderly included in these strategies, and ensuring that vulnerable individuals are included in dialogues and decision making.

Throughout the process of program implementation, SSH4A incorporated continuous monitoring, comparative studies, formative research, learning from monitoring data (qualitative and quantitative), structured knowledge and learning process and adaption with stakeholders. Important activities included events for all program leads and selected stakeholders, field activities, and performance monitoring. 

### 2.3. Pre-study Context

Prior to the intervention, many of the program countries had existing government-supported CLTS activities taking place, and in many cases SNV was involved in those pilots. Countries also had, to some extent, efforts to improve supply-chain [17,18]. However, at the time, CLTS was implemented at the village level and while successful in a number of villages, there was no clear road to scale or sustainability. Many stakeholders sought the solution in improvements of CLTS itself, but with SSH4A, SNV wanted to go beyond CLTS-centered programming for rural sanitation.

Stalled progress and slippage were important problems that SSH4A hoped to address. Stalled progress occurred in many of these countries for various reasons, including remaining villages are often more rural and difficult to reach with CLTS campaigns, late adopters are often less interested in the interventions, quality of triggering, supply chain limitations, and scaling strategies and both human and monetary resources are sometimes inadequate [18]. Slippage has also been a considerable problem as CLTS interventions often result in unimproved or low durability latrines [19]. The SSH4A approach implements CLTS-like activities through their demand creation, but the multi-dimensionality of the other three intervention components is meant to address barriers and more effectively scale sanitation interventions in a variety of contexts. 

### 2.4. Data Collection and Follow-Up

Data were repeated cross-sectional in nature, collected at baseline (June 2014), followed by Round 2 (between December 2015 and January 2016, Round 3 (January 2017) and Round 4 (January 2018). SNV began implementing SSH4A following the baseline survey. 

A multi-stage cluster sampling technique was used to select a random and representative sample. In the first stage, a random sample of sub-districts or districts was selected, with their selection probability proportional to the population size. The second sampling stage was villages/towns within each district/sub-district selected using random sampling proportional to size. Households were then randomly sampled from each village/town, and census data were used to produce sampling weights. The households recruited into the study are not necessarily the same persons at each time point. The study took place only in areas that were considered rural by their respective countries. 

Household surveys were collected using Akvo FLOW mobile application software with surveys standardized across all 11 countries [20]. Questions on the household surveys were structured into modules, which includes questions on the household, household members, household wealth, sanitation, sanitation use, hand washing, and direct observations of WASH facilities.

SNV received approval from each of the individual countries to collect the data. Data were collected by trained enumerators from the heads of households and/or the adult members of the sampled households. The inclusion criteria required the respondent be 18 years old or above and the household to be within the program area. SNV used standard informed consent process for every survey, and all data were protected and secured. The study authors were later engaged by SNV as external evaluators to complete the study and were provided deidentified data. The Institutional Review Board of Emory University deemed the study exempt from review. The analysis framework was determined at the start of the study, prior to any data analysis.

### 2.5. Outcome Variables

The primary outcome was program-level coverage of at least basic sanitation, which we defined by the Joint Monitoring Program for Water Supply and Sanitation (JMP) to mean having an improved sanitation facility that is not shared with other households [21]. Secondary variables of interest include sanitation type, handwashing facility access, and safe disposal of child feces. Safe disposal of child feces was assessed only among the subset of households that had a child less than 3 years old. We assessed toilet type using structured observations in the household. Because some toilet types were extremely rare, the toilet type variable was categorized into four levels. These include no toilet, an unimproved toilet (e.g., hanging toilet, pit latrine without a slab and other toilet types), and improved latrine, (e.g., composting toilet, ventilated improved pit, pit latrine with a slab), or a flush/pour toilet. To characterize access to handwashing facilities, we use a binary variable assessing whether or not soap and water were both available at the handwashing station near the latrine within 10 paces. We also asked households to recall promotional hygiene activities that were promoted by the local government with support of SNV, and we report these changes over time. In households with children under three years of age, we assessed safe disposal of child feces, measured by self-report.

### 2.6. Equity Across Vulnerable Groups

We assessed the primary outcome, but stratified by several different equity variables of interest: wealth quintiles, households with elderly, female-headed households, and disability within the households. We use the word equity to mean similarities in the levels of coverage when comparing vulnerable and non-vulnerable groups. Beginning in 2014, the household surveys included questions about household assets based on parameters included in each country’s Demographic and Health Survey demographic questions. Using the data collected, wealth indices were created for each household. Each household was categorized as being within a specific wealth quintile based on national-level wealth index cutoffs, which were estimated using the EquityTool developed by the Social Franchising Metrics Working Group (https://www.equitytool.org/development/). In the analyses, the lowest two wealth quintiles and highest two wealth quintiles were used as a proxy to denote households of lower socioeconomic status and higher socioeconomic status respectively [22]. We assessed whether each household was headed by a female or male based on participant responses. We also assessed whether there were persons with disability in the household, measured by questioning whether participants reported that they encountered “a lot of difficulty or were unable to: (1) see, (2) walk or climb steps, or (3) perform self-care such as washing or dressing”. These questions were adapted from the Washington Group short set of disability questions [23]. Any person 55 years or older was considered an elderly person [24] Households that were female-headed, within the lowest two wealth quintiles, and/or with at least one elderly person or disabled person were considered “vulnerable” for the purpose of this study. 

### 2.7. Analysis

Data are presented as percentage point, rather than percent changes since countries had dramatically different baseline characteristics. Program-level descriptive statistics (percentages and averages) for each of the key outcomes were reported by data collection round. We also compare the difference in the prevalence of the outcome variables between baseline and follow-up rounds. We accounted for the stratified design and applied sampling weights to ensure representativeness with the program areas. Data were cleaned using STATA 14 SE (Stata Corp., College Station, TX, USA) and analyzed using STATA and SAS version 9.3 (SAS Institute, Cary, NC, USA).

### 2.8. Synthesis of Results Across Countries

We used meta-analyses to calculate a pooled effect, measuring the absolute change in latrine coverage across all of the different intervention sites. To obtain a pooled estimate that accounted for the varying sizes of the program areas, we weighted by the population sample size. We used forest plots to present these differences in coverage both by study site and overall. 

### 2.9. Equity Analyses

Analyses similar to those described for the primary outcome above were done, but stratifying to compare the equity of sanitation or toilet type variables between vulnerable and non-vulnerable groups. We used a similar model to that used for the primary outcome analysis above, but additionally stratified to compare the equity of the outcome variables between the vulnerable and the non-vulnerable groups. We created a model where we introduced interaction terms between the vulnerability variable and study round and used a difference-in-difference approach to assess equity of sanitation coverage over time; this approach compares the increases in coverage over time between the vulnerable and the non-vulnerable groups.

### 2.10. Multivariable Analyses

Due to concerns of confounding, we also do a sensitivity analysis using a multivariable regression model, controlling for all of the demographic predictors simultaneously. 

## 3. Results

### 3.1. Demographics

23,805 people were surveyed who represent the 8,318,801 people living in the program areas at baseline (the population grew to about 8.7 million at the final follow-up, even excluding South Sudan; Table 1). SNV was also working in some additional places whose populations were not included in the study due to SNVs inability to safely there over time (see Appendix A Text 1). The average number of households surveyed each round was 21,411 households. The proportion of female headed households and households with a person with disabilities was low (Table 1). Bhutan, Ghana, Kenya, South Sudan, Nepal 1 and Nepal 2 had higher proportions of households with any elderly members living there as compared to Ethiopia, Indonesia, Mozambique, Tanzania, Uganda and Zambia.

The number of household members was also usually higher in these same countries that had elderly in the household, representing a trend for extended family living together. Mozambique, South Sudan and Tanzania reported a lower proportion of households within the lowest two wealth quintiles.

### 3.2. Sanitation

**Figure A1:**
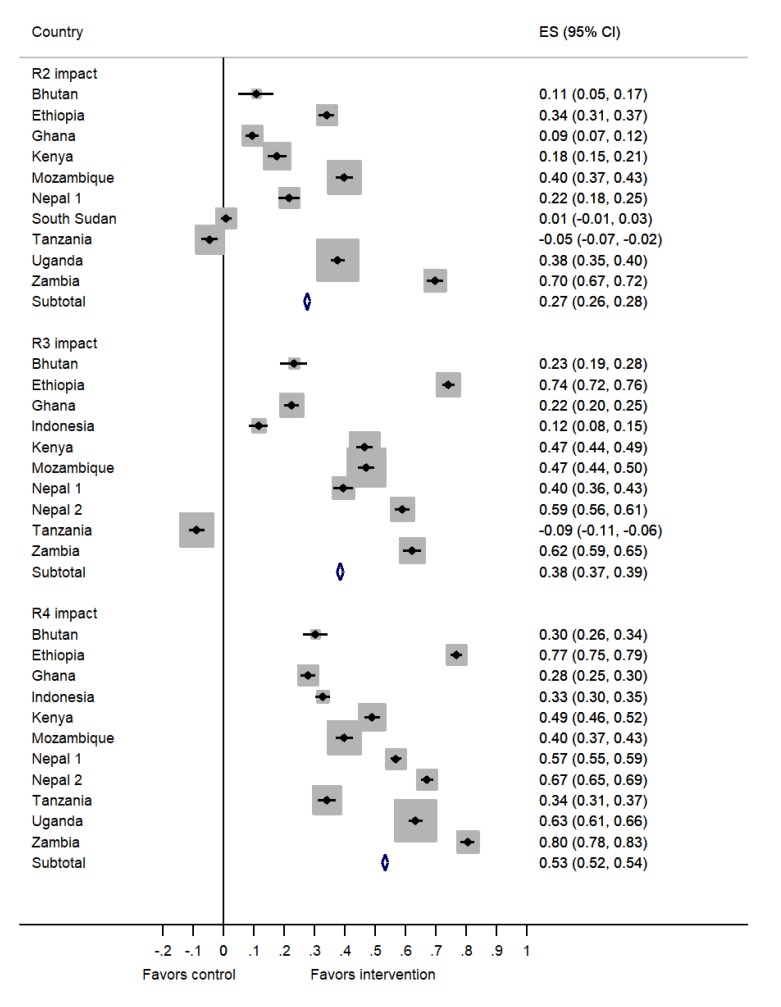
Forest plot showing the impact of the SSH4A interventions on basic latrine coverage, weighted by population size. ES—estimate of prevalence difference compared to baseline.

Nearly all program areas had very low basic sanitation coverage levels prior to program implementation, with only Bhutan and Indonesia having baseline coverage levels greater than 50% (Table 2). There were appreciable gains in sanitation coverage across all program areas, except South Sudan. Meta-analysis results showed a 53-percentage point (95% CI: 52%, 54%) increase in the presence of basic sanitation from baseline to endline (see Figure A1). In most cases, countries that had large increases in the prevalence of sanitation had correspondingly large estimated increases in the total population gaining toilet access (e.g., Ethiopia, Kenya, Mozambique, Nepal, Uganda, Zambia; Table 2). Ghana and Bhutan had similar increases in the prevalence of sanitation over time (+28% and +30%, respectively), but the estimated population that gained access to a toilet was about 10 times higher in Ghana than Bhutan as the population in Ghana was bigger. Overall, we estimate 4,800,711 people gained access to basic sanitation between baseline and the final follow-up. 

**Figure 1:**
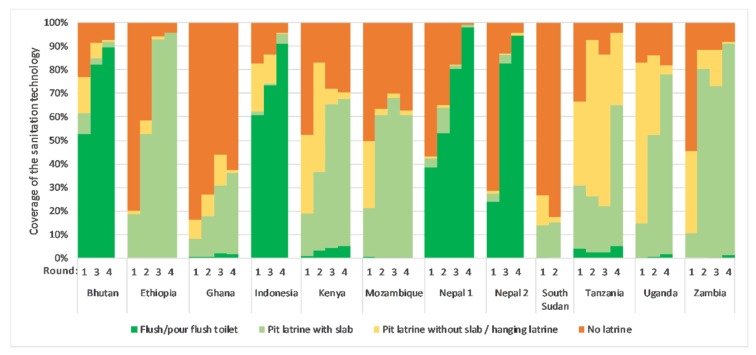
Shift in the coverage of various sanitation technologies across the four rounds.

Countries progressed up the sanitation ladder in different ways (Figure 1). Eight out of 12 sites had continual increases in coverage across the entire study period. Zambia and Mozambique had initial increases in coverage that then plateaued, whereas Tanzania saw an initial plateau that then a later had increases in coverage. South Sudan did not have an increase in coverage from round one to two, and then the study was discontinued there due to instability in the region. Generally, we observed that South and East Asian countries (Bhutan, Indonesia, Nepal) progressed up the sanitation ladder through the implementation of flush/pour flush toilets, while African countries implemented pit latrines with slabs. Several countries (e.g., Tanzania and Uganda) appeared to be replacing unimproved sanitation with improved latrines, whereas other countries like Ethiopia replaced no sanitation at all with improved sanitation. When assessing progress at each of the four follow-up points, most countries saw improvements in basic sanitation over time, although the trends in how that progress happened sometimes varied (Figure 1 and Table A1).

We performed a sensitivity analysis to compare the SSH4A sanitation coverage changes to nationally reported JMP results in each country [25]. While the SSH4A study sites had similar baseline prevalences of basic sanitation to those prevalence reported by the JMP in 2014, the JMP-reported gains in coverage between 2014 and 2017 were modest in these countries (Table 3) compared to the SSH4A results. The largest JMP-reported increase in basic sanitation coverage was a 9% increase in coverage in Nepal, and the least progress was made in the African countries, where four of the African countries had increases in coverage of 0% or less (Table 3). 

### 3.3. Disposal of Child Feces

At baseline, only two out of 12 program areas—Tanzania and Uganda—had more than half of their households safely disposing of child feces (Table 4). Disposal of child feces in Bhutan and South Sudan at final follow-up were not meaningfully different than at baseline (Bhutan: −4%, 95% CI:−16%, 9% and South Sudan: +3%, 95% CI: 0%, 7%). All other program areas had higher prevalence of safe disposal of child feces at the final follow-up than at the baseline visit (differences ranged from +21 to +81 percentage points). Ethiopia had the highest gains in safe disposal of child’s feces (+81%, 95% CI: 78%, 84%), followed by Nepal 1 (+59%, 95% CI: 54%, 64%), and Zambia (+56%, 95% CI: 51%, 61%).

### 3.4. Hygiene

At baseline, the observed prevalence of handwashing facilities with soap and water was low across most of the program areas (Table 5). Only Bhutan and Indonesia had baseline presence of handwashing stations greater than 10% (33% and 16%, respectively); these two countries were also more likely to have had piped water at baseline. Considerable gains were seen over time in the Nepal sites (+69% and +70%) and Tanzania (+34%), with smaller gains in most other countries. 

### 3.5. Equity of Basic Sanitation between Vulnerable and Non-Vulnerable Groups

There were very few differences in equity of coverage while comparing the change in sanitation coverage between vulnerable and non-vulnerable households (Table 6 and Table A2). Specifically, the increase in sanitation coverage over time was similar for female headed vs. male headed households (+0.5%; 95% CI: −1.7%, 2.6%), and also for households with disabled members vs. households without any disabled members (0.0%, 95% CI: −4.3%, 4.4%). Households with elderly members were more likely to have gained sanitation over time compared to households without any elderly (+3.2%; 95% CI: 1.3%, 5.0%). Conversely, households in the lowest two socio-economic status (SES) quintiles were less likely to have gained sanitation coverage over time as compared to households within the highest two wealth quintiles (−5.3%, 95% CI: −7.5%, −3.1%). The sensitivity analyses using multivariable analyses to simultaneously control for other demographic predictors showed results that were similar to unadjusted models (results not shown). The country-level data revealed similar findings compared to the aggregate data in that there were SES disparities at the final round of the program across many countries (see Table A3). 

**Figure 2:**
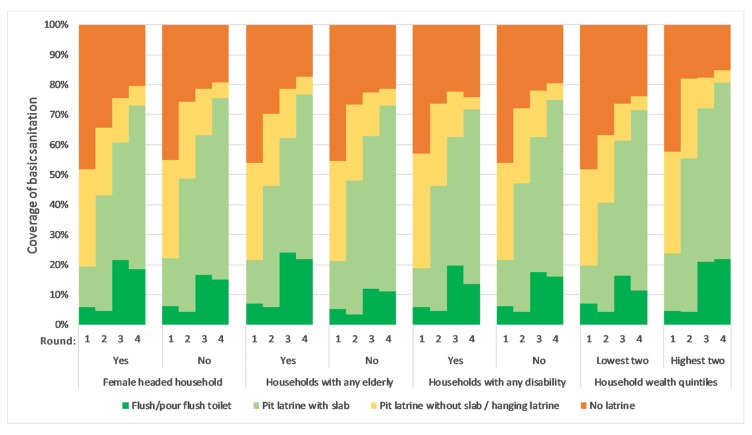
Equity in sanitation technologies over the four rounds, aggregated across all countries.

Figure 2 shows that both vulnerable and non-vulnerable groups advanced up the sanitation ladder over time, but also shows that at any given point in time the toilet types being used tended to be similar when comparing vulnerable and non-vulnerable groups. The toilet type trends were very similar when comparing female headed and male headed households and when comparing households with and without persons with disability. Toilet types for households with an elderly person versus households with no elderly members were also similar, except that households with elderly members were more likely to have flush/pour flush toilets in the later rounds. Higher SES households were more likely to improve their latrine coverage and flush/pour flush toilet coverage over time, although the lower SES households still showed improvements over time. 

## 4. Discussion

Our analysis found that the SSH4A approach increased sanitation coverage and progress up the sanitation ladder across a variety of countries and contexts. When examining equity of the SSH4A approach, we found that sanitation coverage increased significantly over time for both vulnerable and non-vulnerable groups. However, there was higher coverage of sanitation among higher SES households. 

The impact of this approach on increasing basic latrine coverage was striking when compared to previous documented programs and interventions. A recent systematic review found that sanitation interventions to date have only increased latrine coverage by an average of 14 percentage points [4]. Five out of 11 countries in the study reported at least 50 percentage points change in coverage of basic sanitation at the end of the SSH4A intervention as compared to only one country of the 27 intervention studies reported in a systematic review by Garn et al. [4]. One possible reason for the success of the SSH4A approach may be the persistent and sustained presence of the interventions over time. Hulland et al. discussed in their review the how important frequent and sustained interaction with intervention personnel is for WASH sustainability [26]. In this evaluation, many of the study sites did not fully realize meaningful gains in sanitation coverage until the later years of this study. It is possible that other programs (e.g., those reviewed in Garn et al. systematic review) may have also realized greater gains had they persisted with the intervention for a longer period of time. This evaluation also primarily took place in areas with low baseline sanitation coverage. Like many other studies [27], it was more difficult for the SSH4A program to reach the last 10% of each study population with toilets as compared to reaching earlier adopters. The findings in this evaluation may therefore not be generalizable to areas with high initial sanitation coverage. 

The multi-dimensional approach may have been important in supporting the success of the intervention, as these separate dimensions might have assisted with addressing the unique barriers of the different program areas. Other sanitation approaches may focus on one single element of sanitation service delivery (e.g., marketing, education, triggering) which may result in less overall improvement since successful sanitation programs are highly dependent on other factors, for example, successful triggering might depend on governance or a marketing components already being in place [28]. Each country’s program did not follow the same model or prioritization of components, but instead tested a range of activities tailored to local contexts which covered a breadth of approaches including: targeting specific areas; developing an outreach strategy based on the locally available structures and organizations, engaging local leadership for mobilizing collective action; tailoring mobilization, BCC and demand creation to local context and groups and encouraging inclusive and pro-poor sanitation business models and technologies. Other activities that were tailored to local contexts involved working with right holder groups, integrating inclusion into government planning and budgeting, evidence-based advocacy, and support self-financing. Specifics on contexts of the individual countries and on country-specific programmatic modifications and emphases are discussed in the appendix (Appendix A Text 1).

The SSH4A approach increased sanitation coverage among vulnerable groups from baseline to the end of the program at similar rates to vulnerable groups. These results contrast to those from other studies, which have found lower sanitation coverage among households with elderly persons, [29,30] among persons with disability, [31] and female headed households [12]. Our analysis found lower coverage of sanitation among lower SES households at endline, which is similar to findings on sanitation coverage and SES reported elsewhere in the literature [32,33,34,35]. The high sanitation coverage achieved might be due to several approaches adopted by the program, specifically with the intent to target and to reach these vulnerable groups. The use of “toolbox” approach of adaptive strategies might better address the varied needs of these vulnerable groups in different contexts, and at different stages of programming facilitated improved sanitation uptake among these individuals. 

There are several limitations to this study. First, there was not an external comparison group. Consequently, we compared final results to the baseline data within the same study areas. This can be problematic if there were additional programs or policies that might have impacted WASH coverage in these areas over time. Our comparison to JMP results indicates that gains observed in the SSH4A program areas happened during a time where there was little national improvement in basic sanitation across these same countries, giving us confidence that the gains detected may be attributable to the program and were not reflective of a broader secular trend. The government engagement component of the SSH4A program would make it very difficult to do a study with an internal control group. 

While we saw considerable increases in sanitation coverage over time, the study did not analyze why and how the SSH4A approach led to considerable sanitation improvements. This is an important component for future study. Another limitation is that the data collection periods across partners were not always aligned. Some WASH indicators are correlated to seasonality, and collecting data at different times of year may make comparison both between partners or between baseline and endline less reliable. The measure used for households with persons with disability was derived from the Washington Group Short Set, but is limited in that it is only a screening tool used to identify people who *may* have disability by asking questions to a household member who is not necessarily the person with the disability. The actual population of individuals with disabilities in households is likely to be higher than that reported in our study. Lack of direct targeting towards households that had persons with disabilities weakens the equity findings, although the large sample size of our study still allowed us to find a substantial number of these household. Another limitation of this study was difficulty to implement the approach and to collect data in South Sudan for all four rounds due to the conflict [36]. While we did not see increases in sanitation coverage in South Sudan over the first two rounds of data collection, this may have been due to a myriad of reasons including population instability due to migration. We were not able to fully assess the sustainability of these interventions. While stalled progress and slippage are important problems that the SSH4A approach hopes to address, answering the question of whether these improvements will be sustained, or if there will be slippage, will require returning to the areas where SNV is no longer working and reassessing the WASH conditions over time. Finally, while the study took place across many countries and contexts, these findings might not be generalizable to all contexts, particularly as the study took place in rural settings. 

## 5. Conclusions

This is one of the first peer-reviewed large-scale evaluations of a rural sanitation program, and is the first published evaluation of the SSH4A approach—an approach that appears to have been successful across a variety of countries and contexts and in reaching vulnerable groups. These data were collected using uniform questionnaires allowing for the comparison of variables between countries. The data are relevant in a global context at a time when there is a lack of understanding about how to best increase sanitation coverage and improve progress up the sanitation ladder. The gains here could serve as a benchmark for other similar programs. However, additional learning would be useful to understand the programmatic and contextual factors that lead to success in implementing sanitation and hygiene interventions such as this one.

## Figures and Tables

**Table 1:** Characteristics of the study population at baseline across 11 countries, 2014 (*n* = 23,805).

Characteristics	Bhutan	Ethiopia	Ghana	Indonesia	Kenya	Mozambique	Nepal 1 ^1^	Nepal 2 ^1^	South Sudan	Tanzania	Uganda	Zambia
Total living in program area ^2^	95,111	454,255	469,964	174,547	816,934	1,267,424	460,873	521,548	487,105	996,535	2,033,442	541,063
Total sampled	751	2167	2112	2039	1953	1888	2979	2492	2131	2177	2055	1061
Female headed household (%) ^3^	214 (25)	490 (23)	274 (17)	197 (10)	428 (23)	451 (24)	554 (16)	372 (15)	736 (33)	427 (20)	473 (23)	282 (26)
Households with any person with disability (%) ^3^	57 (7)	53 (2)	226 (12)	89 (5)	227 (11)	71 (4)	410 (10)	226 (9)	187 (8)	353 (17)	220 (12)	98 (9)
Households with any elderly (%) ^3,4^	459 (62)	849 (39)	1431 (70)	978 (48)	1101 (56)	461 (24)	1749 (60)	1572 (65)	1259 (61)	1071 (49)	956 (49)	421 (39)
Households wealth quintiles ^5^												
Lowest two quintiles (%)	163 (44)	851 (39)	773 (36)	1046 (47)	1384 (68)	519 (28)	1990 (57)	1269 (51)	86 (4)	551 (23)	790 (40)	429 (40)
Middle quintile (%)	121 (33)	449 (21)	466 (22)	530 (27)	288 (17)	440 (23)	761 (32)	976 (39)	433 (20)	925 (43)	412 (20)	229 (22)
Highest two quintiles (%)	86 (23)	867 (40)	873 (41)	463 (25)	281 (16)	929 (49)	228 (11)	247 (10)	1612 (76)	701 (33)	853 (40)	403 (38)
Number of HH members, mean (SD)	4.6 (0.08)	4.8 (0.05)	10 (0.16)	4.1 (0.04)	8 (0.16)	4.2 (0.06)	6.8 (0.06)	7.1 (0.08)	7.3 (0.09)	7.3 (0.1)	7.9 (0.31)	5.4 (0.09)

^1^ There were two separately funded program areas in Nepal, which we call Nepal 1 and Nepal 2. ^2^ Population size of the entire program catchment areas at baseline. ^3^ Sampling weights were used so the percentages are representative of the program areas. ^4^ Any persons ≥50. ^5^ These the percent of people in the poorest two wealth quintile based on national wealth assets. HH—households; SD—standard deviation.

**Table 2:** Change in coverage of basic sanitation, shown by program area.

Program Area	Baseline Sanitation Prevalence (95% CI)	Final Follow-up Sanitation Prevalence (95% CI)	Baseline to Final Difference (95% CI)	Estimated Population that Gained Toilet Access
Bhutan	62% (58%, 65%)	92% (90%, 94%)	+30% (26%, 34%)	28,835
Ethiopia	19% (17%, 20%)	95% (95%, 96%)	+77% (75%, 79%)	422,469
Ghana	8% (7%, 10%)	36% (34%, 38%)	+28% (25%, 30%)	146,331
Indonesia	62% (60%, 65%)	95% (94%, 96%)	+33% (30%, 35%)	56,309
Kenya	19% (17%, 21%)	68% (66%, 69%)	+49% (46%, 52%)	446,967
Mozambique	21% (19%, 23%)	61% (59%, 63%)	+40% (37%, 43%)	608,361
Nepal 1	42% (41%, 44%)	99% (99%, 100%)	+57% (55%, 59%)	283,219
Nepal 2	27% (26%, 29%)	94% (94%, 95%)	+67% (65%, 69%)	375,077
South Sudan	14% (13%, 15%)	15% (14%, 16%) ^1^	+1% (−1%, 3%) ^1^	6452 ^1^
Tanzania	31% (29%, 33%)	65% (63%, 67%)	+34% (31%, 37%)	390,957
Uganda	15% (13%, 17%)	78% (77%, 79%)	+63% (61%, 66%)	1,480,076
Zambia	11% (9%, 13%)	91% (90%, 92%)	+80% (78%, 83%)	555,658

^1^ The final follow-up in South Sudan was after one year (i.e., round 2). CI—confident interval.

**Table 3:** Joint Monitoring Program for Water Supply and (JMP) reported change in coverage of basic sanitation, shown by country.

Country	JMP Basic Sanitation Coverage in 2014	JMP Basic Sanitation Coverage in 2017	JMP Basic Sanitation Difference
Bhutan	66%	69%	+3%
Ethiopia	7%	7%	0%
Ghana	16%	18%	+2%
Indonesia	68%	73%	+5%
Kenya	30%	29%	−1%
Mozambique	26%	29%	+3%
Nepal	53%	62%	+9%
South Sudan	9%	11%	+2%
Tanzania	3%	5%	+2%
Uganda	18%	18%	0%
Zambia	26%	26%	0%

**Table 4:** Reported safe disposal of child feces, shown by program area (restricted to households with children <3 years of age).

Program Area	Baseline Safe Disposal Prevalence (95% CI)	Final Follow-up Safe Disposal Prevalence (95% CI)	Baseline to Final Difference (95% CI)
Bhutan	37% (29%, 46%)	34% (25%, 43%)	−4% (−16%, 9%) ^1^
Ethiopia	16% (13%, 19%)	97% (96%, 99%)	+81% (78%, 84%)
Ghana	10% (7%, 13%)	47% (44%, 50%)	+37% (33%, 41%)
Indonesia	49% (42%, 56%)	79% (74%, 84%)	+ 30% (22%, 39%)
Kenya	35% (32%, 39%)	69% (66%, 72%)	+34% (29%, 38%)
Mozambique	43% (40%, 47%)	68% (63%, 72%)	+24% (18%, 30%)
Nepal 1	28% (25%, 30%)	87% (83%, 91%)	+ 59% (54%, 64%)
Nepal 2	20% (17%, 23%)	69% (66%, 73%)	+49% (44%, 54%)
South Sudan	21% (19%, 23%)	25% (22%, 27%)	+3% (0%, 7%) ^1^
Tanzania	61% (58%, 64%)	96% (94%, 97%)	+35% (31%, 38%)
Uganda	71% (68%, 75%)	92% (91%, 94%)	+21% (17%, 25%)
Zambia	38% (33%, 42%)	94% (92%, 96%)	+56% (51%, 61%)

^1^ Variable was unavailable from Bhutan and South Sudan for the final visit. Round 2 is shown for South Sudan, and Round 3 is shown for Bhutan.

**Table 5:** Access handwashing (HW) stations with soap and water by program area.

Program Area	Baseline HW Station Prevalence (95% CI)	Final Follow-up HW Station Prevalence (95% CI)	Baseline to Final Difference (95% CI)
Bhutan	33% (29%, 37%)	65% (62%, 69%)	+32% (27%, 37%)
Ethiopia	0% (0%, 0%)	26% (24%, 28%)	+26% (24%, 28%)
Ghana	0% (0%, 1%)	11% (10%, 12%)	+11% (10%, 12%)
Indonesia	16% (14%, 18%)	36% (34%, 39%)	+20% (17%, 23%)
Kenya	1% (0%, 1%)	10% (9%, 11%)	+9% (8%, 10%)
Mozambique	4% (3%, 4%)	16% (15%, 18%)	+13% (11%, 14%)
Nepal 1	8% (7%, 9%)	77% (74%, 79%)	+69% (66%, 72%)
Nepal 2	6% (5%, 7%)	76% (75%, 78%)	+70% (68%, 72%)
South Sudan	2% (2%, 3%)	1% (1%, 1%) ^1^	−1 (−2%, −1%) ^1^
Tanzania	0% (0%, 1%)	35% (33%, 37%)	+34% (32%, 37%)
Uganda	1% (0%, 1%)	4% (3%, 4%)	+3% (2%, 4%)
Zambia	0% (0%, 0%)	24% (22%, 26%)	+23% (21%, 25%)

^1^ The final follow-up in South Sudan was after one year (i.e., round 2).

**Table 6:** Change in coverage of basic sanitation over time, compared between vulnerable and non-vulnerable groups. Data are aggregated across all countries.

Characteristics	Baseline Sanitation Prevalence (95%CI)	Endline Sanitation Prevalence (95%CI)	Baseline to Endline Difference (95% CI)	Difference in Differences (95% CI)
Female headed households				
Yes	19% (18%, 21%)	73% (72%, 74%)	54% (52%, 56%)	0.5% (−1.7%, 2.6%)
No	22% (21%, 23%)	75% (75%, 76%)	53% (52%, 54%)	
Households with any elderly				
Yes	22% (21%, 23%)	77% (76%, 78%)	55% (54%, 56%)	3.2% (1.3%, 5.0%)
No	21% (20%, 22%)	73% (72%, 74%)	52% (51%, 53%)	
Households with any disability				
Yes	19% (17%, 21%)	72% (68%, 76%)	53% (49%, 57%)	0.0% (−4.3%, 4.4%)
No	22% (21%, 22%)	75% (74%, 76%)	53% (52%, 54%)	
Socioeconomic status				
Lowest two wealth quintiles	20% (19%, 21%)	72% (71%, 73%)	52% (51%, 53%)	−5.3% (−7.5%, −3.1%)
Highest two wealth quintiles	24% (22%, 25%)	81% (80%, 82%)	57% (56%, 59%)	

## Appendix A

Text 1. Area specific SSH4A details.

*Bhutan*. The goal of the Bhutanese government is to increase access to universal sanitation coverage by 2023 [37]. Since the royal decree of 1992, subsidies are no longer included in sanitation programs in Bhutan in order to promote self-reliance, sustainability, and affordability [38,39]. SNV is the main collaborating partner of the Royal Government of Bhutan in terms of rural sanitation and hygiene strategies and activities with high-level government officials working hand-in-hand with SNV specialists. This collaboration with the national government has led to great improvements in sanitation coverage through the SSH4A approach. From the study onset, Bhutan has focused on the implementation of flush/pour flush toilets, which requires not only creating demand but also stronger emphases on sanitation supply chain strengthening through developing linkage between potential suppliers and the communities/households. However, as many rural households cannot afford pour flush toilets, do-it-yourself (DIY) pit latrine building pamphlets have been developed by SNV to help poor households using local, free (or inexpensive) materials. As large-scale marketing is not feasible in Bhutan, the supply chain in terms of product development is not the main focus of the program. Instead, local masons are trained on how to build improved latrines including orientation of health workers on critical aspect of toilet construction to reach out to households and communities individually. The private sector is also included in demand creation triggering. We are evaluating the implementation of the SSH4A approach in the Samtse and Tashigang districts with around 95,111 people living in the study areas.

*Ethiopia*. Water supply, sanitation and hygiene in Ethiopia are addressed as integrated packages, and the government is committed to implementing a Sector Wide Approach (SWAp) through the ONE WASH National Programme, supported by a number of Development Partners and NGOs. The government has sets out its development goals in successive Growth and Transformation Plans (GTPs), which identify water, sanitation and hygiene as priority areas for achieving sustainable growth and poverty reduction. In line with the second GTP, the Ethiopian Government adopted the Universal Access Plan (UAP). To facilitate achievement of the GTP and UAP targets, the government has prepared a WASH Implementation Framework (WIF) to provide guidance for implementing the program and also defines the roles and responsibilities of major stakeholders in the WASH sector. Moreover, Health Sector Development Programmes (HSDP l, ll, lll and lV) in line with the Plan for Accelerated and Sustained Development to End Poverty (PASDEP), and the Growth and Transformation Plan of 2011–2015 (GTP I) and 2015–2020 (GTP II) have been introduced to address the water, hygiene and sanitation problems of the country. One of the main innovations of the HSDP has been the Health Extension Programme (HEP) that aims to reach universal coverage of primary health care and improve the quality of health services in rural areas and partly in the urban areas including sanitation and hygiene. The rural sanitation approach employed by the government is Community Led Total Sanitation and Hygiene (CLTSH) after developing National CLTSH implementation and verification guideline since 2010 together with WASH development partners in the country.

The SSH4A approach in Ethiopia included strengthening and using the existing government and community structures for demand creation, sectoral alignment and behavior change communication towards sanitation and hygiene; institutionalizing BCC with the lead agencies being the health and education sectors; revitalizing zonal, *woreda* and *kebele* WASH teams from different sectors to coordinate WASH activities and verifying ODF at each level; and establishing and strengthening Sanitation Marketing Centres and artisans in all six districts to produce and provide sanitation and hygiene products and services to the community with affordable costs during four years of the project period. SSH4A was implemented in 6 *woredas*, with approximately 454,255 people living in the study areas.

*Ghana*. Similar to Ethiopia, in Ghana the national government has named CLTS as one of the preferred rural sanitation approach, and the local government at Metropolitan and Municipal District Assembly level is responsible for the implementation of sanitation related interventions [19]. CLTS as a standalone strategy has not been effective across all contexts in terms of sustaining coverage, and has generally resulted in unimproved latrines with low durability. An emphasis of the SSH4A approach in Ghana was behavioral change communication using multiple channels and methods of outreach, with the support of stakeholders [40]. The national government worked with several iNGOs to develop a BCC manual that can be applied to specific local context based on the needs of different cultural and social groups. Messages and materials are delivered by local government workers. BCC campaigns focus both on adopting toilet use and hygienically cleaning and maintaining latrines especially in schools. The program also emphasized providing a wide range of low-cost sanitation options including SAFI latrine (a product of an action research conducted by SNV in Ethiopia, Kenya and Tanzania), and training including entrepreneurial skills and capacity development of government staff and artisans to help raise community awareness on reliable sanitation options. It also focused on buy-ins from local government and the Traditional Authority who were used as influential persons in steering demand creation and scaling uptake of sanitation. To address vulnerable populations, the national government developed a pro-poor policy. Local government units are working to adapt and apply this policy as appropriate. Additionally, community support is essential in reaching ODF status as young men typically work together to help vulnerable households build latrines. SSH4A was implemented in 7 districts, with approximately 469,964 people living in the study areas. After the baseline data collection, implementation and data collection began in one additional district, but this district was not included in our study to ensure identical program areas across each of the four rounds.

*Indonesia*. Due to the abundance of water sources in the island targeted by the intervention, Sumatra, the locally preferred technology of choice is the flush/pour flush toilet. The SSH4A intervention was monitored in 5 sub-districts, with approximately 174,547 people living in the study area. Due to the duration and the funding set up in this area, no data collection took place during the second round, so we only present rounds one, three, and four. Similar to Bhutan, the SSH4A approach in Indonesia included a focus on triggering local demand for flush/pour flush toilets, which required emphases to strengthen the sanitation supply chain accordingly.

*Kenya*. The Kenyan constitution declares that it is a basic human right to have access to a reasonable standard of sanitation [41]. Sanitation is a devolved function and the 47 county governments of Kenya play a critical role in accelerating access to basic sanitation in the country. Appropriate policies, strategies and guidelines have been developed at national level to drive this acceleration and several development partners have supported various sanitation and hygiene interventions. The SSH4A approach in Kenya aims at ending open defecation, stimulating business people to offer affordable toilets, encouraging communities to maintain safe hygiene practices and supporting the County Governments to fulfil the constitutional right to a reasonable standard of sanitation. The program worked with 555 promoters and reached 2344 villages. CLTS and BCC were used for demand creation, while artisans were engaged in the production and sale of latrine and handwashing options. The SSH4A was implemented in 11 Sub Counties in Kenya, with approximately 816,934 people living in the program areas.

*Mozambique*. The Mozambican constitution declares that it is a basic human right to have access to a reasonable standard of sanitation, but historically it has been a challenge to increase coverage while the political priority has been for increasing access to water. In 2006, Mozambique experienced a cholera pandemic and needed to develop a strategy that could help communities improve sanitation access rapidly, the National Government named the CLTS approach as the preferred rural sanitation approach and started a one-year campaign named “*Latrina Para Todos*” to push for sanitation facilities construction involving all levels of government. However, since this initial push, national consensus on sanitation approach, prioritization and rates of access have plateaued. Primary emphases of the SSH4A approach in Mozambique included supporting communities in the construction of practicable low-cost sanitation options, using locally available materials and resources, and running BCC campaigns for HWWS and latrine construction via multiple channels. Since 2014, the SSH4A program started early consensus building towards CLTS as the main approach for rural sanitation with relevant stakeholders and trained government district staff to raise community awareness on affordable sanitation options and hygienic use of toilets [42]. SSH4A was implemented in five districts in Nampula province, with approximately 1,267,424 people living in the program areas.

*Nepal*. Nepal’s nationwide sanitation campaign is characterized as a social movement which has a strong political commitment and leadership from the government to achieve Universal sanitation coverage. The country has made steady progress despite changes in governance structures under the new federal system and natural disasters in the period [43]. The National Sanitation and Hygiene Master Plan (NSHMP) launched in 2011, sets clear guidelines for sanitation promotion based on no-subsidy principles while encouraging locally managed financial support mechanisms for potentially vulnerable groups. The Master Plan also sets clear criteria for an improved latrine following the JMP definition with a permanent sub-structure, which has incentivized households to make a one-time investment in durable toilets. More recently, the Constitution of 2015 enshrines access to water and sanitation as a fundamental right, and this has been used by development actors to encourage the elected local governments in supporting progress on sanitation.

The SSH4A approach in Nepal established a strong base for governance by supporting the formation and/or strengthening of multi-stakeholder WASH Coordination Committees at the sub-national, district, and local levels (guided by NSHMP 2011). These platforms have been critical for developing clear strategic guidance, targets, and coordination amongst development partners and different government agencies, as well as political representatives, NGOs, media, and the private sector. At the local level, these platforms have translated the district strategies into sanitation action plans and mobilized the whole community and interest groups around them with a voice for women, people with disabilities, and low-caste groups not only to achieve open defecation free status but of sustaining behaviors and moving towards the next milestone of “total sanitation” with six indicators on sanitation and hygiene. The SNV team adapted CLTS triggering tools and implemented a range of post-triggering strategies to motivate the diverse set of communities to invest in their own toilets. Notably, “political triggering” was used to successfully address the subsidy mind-set of the political cadre, while balancing provision of localized transparent and targeted support to the poorest strata. The team used evidence-based BCC to conduct rigorous BCC campaigns with multiple tools and multiple channels; critically, these started soon after triggering on toilet construction. Capacitated ring producers, masons, and hardware suppliers were linked to communities during demand creation to support selection of affordable and suitable technologies including for flood-prone areas. The SSH4A approach was administered in two different program areas in Nepal (that we call Nepal 1 and Nepal 2 throughout). SSH4A was implemented in eight districts in Nepal 1 and in seven districts in Nepal 2, with approximately 460,873 and 521,548 people living in these catchment areas, respectively. Both projects included districts from the three ecological zones of the country (mountain, hill, and terai). Due to the earthquake in Nepal in 2015, no data collection took place during the second round in Nepal 2, so we only present rounds one, three, and four.

*South Sudan*. South Sudan’s national government has adopted the CLTS approach in rural areas. In spite of this national prioritization, the participation, capacity, and performance of local government in steering this demand creation is low. Success of CLTS is aggravated by a higher presence of humanitarian organizations that subsidize or fully provide latrines, making promotion for self-reliance more difficult. In South Sudan, only the baseline and first follow-up data collections occurred, as SNV could no longer work in the country due to civil unrest. During those two rounds of data collection, SNV attempted to implement the SSH4A approach in two counties, with about 487,105 people living in the study areas. The program put significant emphasis on training local government officials on CLTS and pioneered the roll out of sanitation policies where applicable. Work was also done around BCC outreach in schools and other public institutions.

*Tanzania*. Responsibilities for sanitation in Tanzania are spread across several government sectors [44] with no cohesive sanitation policy [45]. However, there is a strong focus on CLTS especially in hard to reach communities. The national government also implements a fine of around $20 (50,000 TZS) for households without latrines which is enforced through random spot checks. Tanzania generally has low levels of open defecation but with high levels of unimproved facilities, and the practice of open defecation has actually increased in Tanzania since 1990 [44]. The government of Tanzania has rolled out the national sanitation campaign (NSC) that aims at increasing access to sanitation in all districts with specific focus on toilet quality. The program had specific intervention areas spread across the country. The SSH4A approach focused on stakeholder mobilizations, commitment and buy-in from local leaders was also sought through quarterly meetings to review progress and monitor issues arising from the program roll quality. The district teams also engaged in developing their capacities in various areas e.g., monitoring demand at scale, developing localized BCC strategies, identifying and establishing support mechanisms for vulnerable households and enacting or rolling out localized sanitation policies [46]. Households also benefited from market-led interventions to increase access to affordable and better latrines. Private sector trainings were provided to various groups of people so that they could engage with the sanitation value chain, to make it accessible and affordable to the local consumers of the sanitation products and services. As mentioned, the Safi toilet was developed by SNV specialists in Tanzania to provide a safe, durable, comfortable, clean, and affordable option for an improved latrine facility. Action research focused on social and business exclusion mechanisms is taking place to further understand why defiant households choose not to build latrines. Poor households and remote, rural villages tend to focus on DIY latrines made from local materials with the only cost being the labor needed to construct the facility. SSH4A was implemented in five districts with approximately 996,535 people living in the study areas which had high levels of open defecation.

*Uganda*. At the beginning of the SSH4A study in Uganda, a CLTS approach led mainly by local NGOs and through District and Lower Governments (DLG/LLG) was already being implemented [47]. Uganda was in need of a scaling strategy that optimized resources and coordinated activities across sectors [48]. The SSH4A approach in Uganda emphasizes community outreach and empowerment, training leaders and stakeholders on toilet quality, engaging with supply chain actors, and disseminating BCC messages (particularly targeting poor households and households with PWD). Participatory hygiene and sanitation transformation approaches were used, especially in areas where CLTS approaches failed [49]. The Mandona approach, an action-oriented effort, was also applied to accelerate ODF status after the initial CLTS triggering and to motivate communities to adopt behavior change by undertaking simple, immediate, and practical actions [49]. The BCC strategy was anchored at the national level through the national handwashing initiative which was being managed by SNV. The initiative was a partnership of all agencies working on sanitation. SSH4A was implemented in 15 districts, with approximately 2,033,442 people living in the study area. Due to insecurity, three program areas were not sampled during the third data collection round, and in order to ensure that our analyses were done in identical program areas over time, results are only shown in Uganda for rounds one, two and four.

*Zambia*. Aligning with the SDGs, the Zambian government has now committed to achieve country-wide open defecation free status by 2030 [37]. The SSH4A approach in Zambia has focused on encouraging households to upgrade their toilets to National Rural Water Supply and Sanitation Program (NRWSSP) compliant ones in order to see the most significant health benefits [50]. During the NRWSSP 2007–2015 the recommended type of toilet in line with MDGs was the adequate toilet which has four parameters – smooth cleanable floors, hole covered by lid, offers privacy and has a hand washing station. The NRWSSP 2019 – 30 is yet to be launched but the draft is in place. Inspired by the SDGs, in the national ODF strategy, the adequate toilet is defined as “a system which hygienically separates excreta from human contact as well as safe reuse/treatment of excreta in situ (on-site), or safe transport and treatment off-site” SSH4A program activities were heavily centered on demand creation, BCC for hygiene, sanitation governance and supply chain development. Sanitation groups were formed to create the demand, whilst SNV trained artisans on quality latrine production to meet the demand. In line with the NRWSSP guidelines, SNV recruited and trained community champions from their respective wards to implement CLTS activities. These were complimented by traditional leaders (Chiefs) who have adopted sanitation and hygiene as one of their priorities in the National House of Chiefs. The local Authority oversees outreach and implementation of rural sanitation programs. There has been positive effects since coordination and focus on sanitation and hygiene within the government was strengthened. The SSH4A program in Zambia also promoted the Adequate Latrine Options, which enabled households to build latrines using local materials for little to no cost. In addition, the SSH4A program established sanitation committees at district, ward and village levels, which facilitated the pooling of resources to enable households to benefit from bulk purchases of sanitation products. Lastly, the government adopted, and is implementing the DHIS II, a mobile to web monitoring system to monitor progress in sanitation and hygiene nationally. Between 2014 and early 2018, the SSH4A program was implemented in four districts with a population of approximately 541,063 people living in the study areas.

**Table A1:** Coverage and change in basic sanitation by program area.

Program Area	R1 Prevalence (95% CI)	R2 Prevalence (95% CI)	R3 Prevalence (95% CI)	R4 Prevalence (95% CI)	R2 Difference (95% CI)	R3 Difference (95% CI)	R4 Difference (95% CI)
Bhutan	62% (58%, 65%)	72% (68%, 77%)	85% (82%, 87%)	92% (90%, 94%)	11% (5%, 17%)	23% (19%, 28%)	30% (26%, 34%)
Ethiopia	19% (17%, 20%)	53% (51%, 55%)	93% (92%, 94%)	95% (95%, 96%)	34% (31%, 37%)	74% (72%, 76%)	77% (75%, 79%)
Ghana	8% (7%, 10%)	18% (16%, 19%)	31% (29%, 32%)	36% (34%, 38%)	9% (7%, 12%)	22% (20%, 25%)	28% (25%, 30%)
Indonesia	62% (60%, 65%)	-	74% (72%, 76%)	95% (94%, 96%)	-	12% (8%, 15%)	33% (30%, 35%)
Kenya	19% (17%, 21%)	36% (34%, 39%)	65% (63%, 67%)	68% (66%, 69%)	18% (15%, 21%)	47% (44%, 49%)	49% (46%, 52%)
Mozambique	21% (19%, 23%)	61% (59%, 63%)	68% (66%, 70%)	61% (59%, 63%)	40% (37%, 43%)	47% (44%, 50%)	40% (37%, 43%)
Nepal 1	42% (41%, 44%)	64% (61%, 67%)	82% (79%, 84%)	99% (99%, 100%)	22% (18%, 25%)	40% (36%, 43%)	57% (55%, 59%)
Nepal 2	27% (26%, 29%)	-	86% (85%, 88%)	94% (94%, 95%)	-	59% (56%, 61%)	67% (65%, 69%)
South Sudan	14% (13%, 15%)	15% (14%, 16%)	-	-	1% (−1%, 3%)	-	-
Tanzania	31% (29%, 33%)	26% (24%, 28%)	22% (20%, 24%)	65% (63%, 67%)	−5% (−7%, −2%)	−9% (−11%, −6%)	34% (31%, 37%)
Uganda	15% (13%, 17%)	52% (51%, 54%)	-	78% (77%, 79%)	38% (35%, 40%)	-	63% (61%, 66%)
Zambia	11% (9%, 13%)	80% (79%, 82%)	73% (71%, 75%)	91% (90%, 92%)	70% (67%, 72%)	62% (59%, 65%)	80% (78%, 83%)

R1, R2, R3 and R4 represent the annual data collection rounds 1, 2, 3 and 4 respectively. R2 difference, R3 difference and R4 difference represent the difference in prevalence of basic sanitation at rounds 2, 3 and 4, and the baseline sanitation prevalence respectively. We did not have data for prevalence of basic sanitation for Ghana and Nepal 2 at round 2, and for South Sudan at round 3 and 4. No data was also available for Uganda at round 3.

**Table A2:** Basic sanitation coverage over time, compared between vulnerable and non-vulnerable groups. Data are aggregated across all countries.

Characteristics	R1 Prevalence (95% CI)	R2 Prevalence (95% CI)	R3 Prevalence (95% CI)	R4 Prevalence (95% CI)
Female headed households				
Yes	19% (18%, 20%)	43% (42%, 44%)	61% (59%, 2%)	73% (72%, 74%)
No	22% (21%, 23%)	49% (48%, 49%)	63% (62%, 64%)	75% (75%, 76%)
Households with any elderly				
Yes	22% (21%, 22%)	46% (45%, 47%)	62% (61%, 63%)	77% (76%, 78%)
No	21% (20%, 22%)	48% (47%, 9%)	63% (62%, 64%)	73% (72%, 74%)
Households with any disability				
Yes	17% (15%, 18%)	47% (43%, 51%)	62% (58%, 66%)	72% (68%, 75%)
No	25% (24%, 26%)	47% (46%, 48%)	62% (62%, 63%)	75% (74%, 76%)
Socioeconomic status				
Lowest two wealth quintiles	20% (19%, 21%)	41% (40%, 42%)	61% (60%, 62%)	72% (71%, 73%)
Highest two wealth quintiles	24% (22%, 25%)	55% (54%, 57%)	72% (71%, 73%)	81% (80%, 82%)

**Table A3:** The difference-in-difference of basic sanitation coverage comparing vulnerable and non-vulnerable groups.

Country	Coverage Change for Female vs. Male Headed HHs	Coverage Change for HHs with Elderly Members vs. No Elderly Members	Coverage Change for HHs With Disabled Members vs. No Disabled Members	Coverage Change for HHs in Lowest Quintiles vs. Highest Two Quintiles
Bhutan	6% (−4%, 15%)	−7% (−15%, 2%)	1% (−14%, 17%)	35% (23%, 48%)
Ethiopia	6% (1%, 11%)	2% (−1%, 6%)	5% (−6%, 16%)	−9% (−13%, −5%)
Ghana	−11% (−18%, −4%)	0% (−5%, 5%)	10% (−2%, 21%)	−9% (−16%, −2%)
Indonesia	−3% (−11%, 5%)	−4% (−9%, 1%)	−1% (−12%, 11%)	60% (56%, 64%)
Kenya	−5% (−11%, 1%)	1% (−4%, 6%)	−7% (−17%, 3%)	15% (8%, 22%)
Mozambique	−6% (−12%, 1%)	−1% (−7%, 6%)	−9% (−53%, 35%)	27% (20%, 33%)
Nepal 1	−31% (−36%, −26%)	2% (−2%, 6%)	−23% (−29%, −17%)	10% (3%, 17%)
Nepal 2	−9% (−15%, −3%)	3% (−2%, 7%)	12% (4%, 20%)	−11% (−18%, −5%)
South Sudan *	−8% (−12%, −4%)	−6% (−10%, −2%)	14% (6%, 21%)	17% (10%, 24%)
Tanzania	19% (12%, 25%)	2% (−4%, 8%)	6% (−9%, 21%)	10% (2%, 18%)
Uganda	−3% (−9%, 2%)	3% (−2%, 8%)	1% (−8%, 9%)	−17% (−22%, −12%)
Zambia	−6% (−11%, 0%)	5% (1%, 10%)	1% (−10%, 11%)	−12% (−17%, −7%)

* South Sudan’s endline was round 2, rather than round 4.
